# An Equation to Estimate PICC Catheter Length in Very Low Birth Weight Infants

**DOI:** 10.1155/jonm/2067769

**Published:** 2026-07-23

**Authors:** Jiaxin Li, Yubing Dong, Qingling Yu, Aifen Cao, Shan Jiang, Yinsui Huang, Xiaoyun Xiong

**Affiliations:** ^1^ Heping Hospital Affiliated to Changzhi Medical College, Changzhi, Shanxi, China; ^2^ Shenzhen Maternity and Child Healthcare Hospital, Women and Children’s Medical Center, Southern Medical University, Shenzhen, Guangdong, China, fimmu.com; ^3^ Guangzhou University of Chinese Medicine, Guangzhou, China, gzucm.edu.cn

## Abstract

**Objective:**

Peripherally inserted central catheters (PICCs) are essential for very low birth weight infants (VLBWIs), but optimal insertion depth remains challenging. Existing estimation methods often yield suboptimal accuracy. This study aimed to develop and validate anthropometric parameter‐based formulas to predict lower extremity PICC insertion depth using the first popliteal crease as an anatomical landmark in VLBWI, seeking to improve first‐attempt success rates and procedural safety.

**Methods:**

A prospective study was conducted in a tertiary NICU, enrolling 329 VLBWIs who underwent lower limb PICC insertion between May 2023 and May 2024. Data collected included sex, target vein for catheterization, body weight, length, head circumference at insertion, and vertical distance from the puncture site to the first popliteal crease. Pearson correlation and linear regression analyses were performed to examine associations between anthropometric parameters and optimal catheter length. Univariate prediction formulas (weight‐based, length‐based, and head circumference‐based models) and multivariate regression models were established. Bland–Altman analysis was used to validate model agreement.

**Results:**

Univariate regression analysis yielded the following: weight‐based model: total insertion depth (cm) = [8.31 + (5 × body weight [kg])] ± PS‐PTC distance, where “+” applies when the puncture site is distal to the popliteal crease and “−” applies when proximal; 8.31 is the regression intercept and five is the regression slope coefficient. Length‐based model: total insertion depth (cm) = [1.13 + (0.35 × body length [cm])] ± PS‐PTC distance; 1.13 is the intercept and 0.35 is the slope coefficient. Head circumference‐based model: total insertion depth (cm) = [2.33 + (0.43 × head circumference [cm])] ± PS‐PTC distance; 2.33 is the intercept and 0.43 is the slope coefficient. Multivariate regression analysis established four combination models, with Model IV (weight + length + head circumference) showing optimal performance.

**Conclusion:**

Anthropometric parameter–based prediction formulas provide precise guidance for lower extremity PICC placement in VLBWI, enhancing placement success and safety. This approach supports individualized care and is especially valuable where ultrasound guidance is limited.

## 1. Introduction

A peripherally inserted central catheter (PICC) is considered a lifeline in the neonatal intensive care unit (NICU) and plays a crucial role in the survival and management of very low birth weight infants (VLBWIs) [1, 2]. However, VLBWI characteristically have small, fragile vessels, delicate skin, and compromised immunity. If a PICC is inserted too deeply, subsequent adjustment increases infection risk, radiation exposure, and the chance of vascular stimulation or injury; if inserted too shallowly and failing to reach the central vein, the risk of medication safety rises [3]. Moreover, reinsertion due to shallow placement can damage the patient’s vessels and increase nurse workload. Bahoush et al. [4] demonstrated that an optimal catheter tip position can reduce the incidence of phlebitis, arrhythmia, and extravasation while prolonging PICC indwelling time. Therefore, improving the first‐attempt success rate of PICC placement is of great importance [5].

Several studies [6, 7] have shown that lower extremity veins are often preferred for PICC placement in VLBWI. Currently, most domestic and international protocols rely on external body measurements for estimating catheter length, but with variable accuracy, resulting in a success rate of 39.13%–53.66% [8, 9]. The most common clinical method is to measure the distance from the anticipated puncture site to the xiphoid process. However, due to natural flexion of neonates, accurate straight‐line measurement is often unachievable, contributing to increased errors. Although the use of ultrasound guidance in PICC procedures has been proposed to facilitate real‐time catheter tip localization and improve the safety and accuracy of insertions, its widespread routine adoption is challenged by requirements for operator expertise, procedure duration, healthcare resource support, cooperation of VLBWI, and disparities in medical resources among countries [10]. Ultrasound equipment is expensive and requires specialized personnel for operation, which limits its widespread use in resource‐limited settings or primary healthcare institutions [11]. Although ultrasound guidance has demonstrated excellent performance in neonatal PICC placement, its wider adoption is hindered by the lack of standardized operational procedures. In certain situations, ultrasound guidance does not fully resolve the issue of accurate catheter length measurement, particularly in neonates with small body size or tiny vessels [12]. Therefore, there is an urgent clinical need to find simple, accurate, and cost‐effective PICC length prediction methods.

In clinical practice, neonatal weight has been widely utilized in various aspects such as medication dosing, estimation formulas for nasogastric and endotracheal tube lengths, and umbilical vessel catheterization [2, 13]. As an important physiological parameter, weight is closely associated with vessel size, length, and body proportion, yet studies specifically addressing its relationship with PICC length are limited. Kim [14] reported a strong correlation (*R* ^2^ = 0.846) between lower extremity PICC length and weight, but most relevant studies [15–17] are retrospective, and the derived formulas are not universally applicable due to variation in puncture sites. The first popliteal crease of the lower limb, as a clear and easily identifiable anatomical landmark, further reduces measurement errors. Evidence suggests its significance for consistent anatomical localization in neonates with strong reproducibility in clinical practice, and its accuracy is not influenced by changes in patient position [16, 17].

To address these limitations, this study prospectively examined the relationship between lower extremity PICC length and key anthropometric parameters—body weight, body length, and head circumference—using the first popliteal crease as a standardized anatomical reference point in VLBWI. This is the prospective cohort study to systematically develop and validate both univariate and multivariate PICC length prediction models based on this anatomical landmark in VLBWI. Body weight emerged as the strongest predictor of PICC insertion depth, and the multivariate model incorporating weight, body length, and head circumference yielded the best predictive performance, with near‐zero systematic bias confirmed by Bland–Altman analysis. The derived formulas offer clinicians a straightforward, objective, and individualized approach to PICC length estimation, with the potential to reduce tip malposition, lower complication rates, and improve first‐attempt success—particularly in NICUs where real‐time ultrasound guidance is not routinely accessible.

## 2. Materials and Methods

### 2.1. Study Design

A prospective study was conducted to establish a formula prediction equation for the insertion depth of peripherally inserted central catheters (PICCs) in VLBWIs. From May 2023 to May 2024, 352 consecutive VLBWI cases who underwent lower limb PICC insertion at the NICU of a tertiary hospital were initially assessed for eligibility.

### 2.2. Study Population

#### 2.2.1. Definition of Appropriate PICC Insertion Length

For lower extremity PICC insertion in VLBWI, the target catheter tip position is defined as placement at or near the cavoatrial junction (CAJ), the anatomical junction between the superior vena cava and the right atrium. On anteroposterior chest radiographs, this position corresponds radiographically to the T9–T11 vertebral level [15, 16]. Therefore, in this study, “appropriate insertion length” refers to the total catheter length required to position the tip within the T9–T11 vertebral range, as confirmed by a chest radiograph performed within 24 h of PICC insertion. Tips above T9 (over‐inserted) that were subsequently adjusted to the T9–T11 range were included in the analysis; tips below T11 (under‐inserted, failing to achieve central venous positioning) were excluded.

#### 2.2.2. Inclusion Criteria

The inclusion criteria were as follows: (1) neonates with birth weight < 1500 g; (2) PICC insertion via lower limb approach; (3) PICC placement within one week of admission; (4) radiographically confirmed appropriate catheter tip position between the T9–T11 vertebral levels, corresponding to the CAJ; and (5) initially over‐inserted catheters (above T9) were subsequently adjusted to the appropriate position.

#### 2.2.3. Exclusion Criteria

The exclusion criteria were as follows: (1) poor catheter tip visualization on chest radiography; (2) vascular anomalies identified by imaging; (3) congenital lower limb malformations; and (4) infants with edema.

### 2.3. Methods

#### 2.3.1. General Data Collection

General data collected included gestational age, sex, mode of delivery, Apgar score at 1 and 5 min, admission weight, admission length, admission head circumference, insertion site, operator, and the time interval between admission and PICC insertion.

#### 2.3.2. Catheter Insertion Procedure

Six certified PICC specialists performed all procedures following standardized protocols. Operators were assigned coded identifiers (1–6), and first‐attempt success rates were compared to assess inter‐operator variability.

Surface measurement technique: With the infant in the supine position and lower limb extended, the distance from the anticipated puncture site to the xiphoid process was measured to estimate the required PICC length.

#### 2.3.3. Measurement and Recording

Anthropometric measurements: For infants who underwent PICC insertion on the day of admission, admission weight, body length, and head circumference were recorded. For all other infants, these measurements were obtained on the morning of the catheterization day, prior to feeding, during routine care.

Anatomical landmark selection: The first popliteal transverse crease was selected as the fixed anatomical reference point for this study. This landmark was chosen for its distinct visibility, consistent bilateral symmetry, and ease of identification at the bedside, which collectively minimize inter‐measurement variability. Importantly, its accuracy is not affected by the physiological lower limb flexion characteristic of neonates, thereby reducing positional measurement error.

**FIGURE 1:**
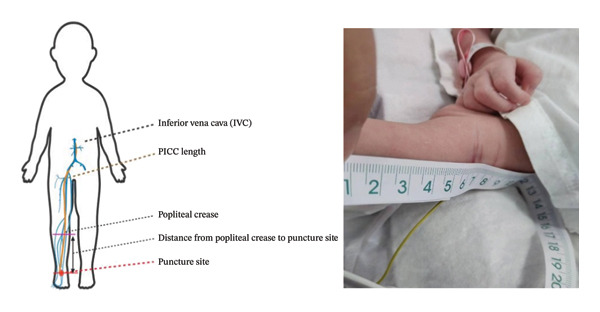
Schematic illustration of the two anatomical distance segments used in this study. The PS‐PTC distance (body surface, measured with a flexible tape measure) represents the perpendicular distance from the venipuncture site to the first popliteal transverse crease. The PTC‐CT distance is a calculated value derived by subtracting the PS‐PTC distance from the actual total PICC insertion length, following radiographic confirmation of catheter tip position at the T9–T11 vertebral level.

Distance measurements: Two anatomical segment distances were defined and measured for each enrolled infant. The puncture site‐to‐popliteal transverse crease (PS‐PTC) distance was defined as the perpendicular body surface distance from the venipuncture site to the first popliteal transverse crease of the ipsilateral lower limb, measured along the medial aspect of the leg with the infant in the supine position and the lower limb fully extended. This measurement was performed after radiographic confirmation of appropriate catheter tip position within the T9–T11 vertebral range on an anteroposterior chest radiograph obtained within 24 h of insertion (Figure 1).

The popliteal transverse crease‐to‐catheter tip (PTC‐CT) distance was not measured directly on the body surface or radiographic image but was derived as a calculated value from two directly obtained measurements: (1) the actual intravascular insertion length and (2) the PS‐PTC distance as defined above. The PTC‐CT distance was calculated as follows: PTC‐CT distance = actual insertion length − PS‐PTC distance when the puncture site is distal to the first popliteal crease, or PTC‐CT distance = actual insertion length + PS‐PTC distance when the puncture site is proximal to the crease. All PTC‐CT values were derived exclusively from cases with radiographically confirmed appropriate catheter tip position, ensuring that the dataset reflects anatomically verified placements only.

Clinical application of prediction formulas: The predicted total PICC insertion depth was calculated by combining the model‐derived PTC‐CT distance with the directly measured PS‐PTC distance according to the following rule:

(1) When the puncture site is located distal to (below) the first popliteal transverse crease: Total insertion depth = PTC‐CT distance + PS‐PTC distance
(2) When the puncture site is located proximal to (above) the first popliteal transverse crease: Total insertion depth = PTC‐CT distance−PS‐PTC distance

All measurements were performed twice by the same trained investigator, and the mean of the two values was recorded to minimize random measurement error.

#### 2.3.4. Data Collection

A dedicated researcher collected all PICC catheterization data, including measured insertion length, radiographically confirmed catheter tip length, puncture site to popliteal crease distance, crease to catheter tip distance, and patient weight, length, and head circumference at time of insertion.

#### 2.3.5. Statistical Analysis

All statistical analyses were performed using SPSS 24.0 software. Categorical variables were summarized as frequencies and percentages for comparing general characteristics among infants with correctly positioned PICCs. Continuous variables were expressed as mean ± standard deviation, with normality assessed using the Shapiro–Wilk test. All continuous variables were confirmed to be normally distributed by the Shapiro–Wilk test (all *p* > 0.05). Pearson correlation analysis was performed to evaluate the associations between PICC insertion length and anthropometric parameters (body weight, body length, and head circumference). Univariate linear regression models were constructed for each anthropometric parameter to develop individual prediction formulas. Multiple linear regression analysis was further conducted to develop a combined prediction model incorporating all three parameters. Model performance was assessed using the coefficient of determination (*R* ^2^). Agreement between predicted and measured insertion lengths was evaluated using Bland–Altman analysis. Statistical significance was set at *p* < 0.05. In the regression equations derived from both univariate and multivariate analyses, the intercept (b) represents the estimated PTC‐CT distance when all predictor variables equal zero, serving as a statistical calibration constant. The regression coefficient (slope, *β*) for each predictor variable represents the expected change in PTC‐CT distance per unit increase in that variable, after controlling for other predictors in multivariate models.

#### 2.3.6. Quality Control

Measurement consistency: A standardized flexible tape measure (single‐use, from the same manufacturer, included in neonatal PICC insertion kits) was used for all measurements. Weight measurement: All weights were obtained using the same model of infant scale; when replacement was necessary, an identical scale from the same manufacturer was used. Operator consistency: PICC measurements and insertions were performed exclusively by fixed PICC team members to ensure standardized procedures. Data collection: Data were collected by dedicated research personnel with dual verification to ensure data accuracy.

### 2.4. Ethics

This study was approved by the Institutional Review Board, approval number: SFYLS[2024]056.

## 3. Results

### 3.1. Participant Baseline Characteristics

**FIGURE 2:**
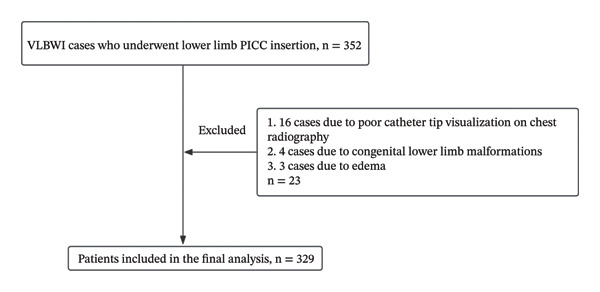
Patient selection flowchart. *Abbreviations:* PICC, peripherally inserted central catheter; VLBWI, very low birth weight infants.

A total of 352 consecutive VLBWI cases who underwent lower limb PICC insertion at the NICU of a tertiary hospital were initially assessed for eligibility. After applying prespecified inclusion and exclusion criteria, 23 cases were excluded for the following reasons: 16 cases due to poor catheter tip visualization on chest radiography, 4 cases due to congenital lower limb malformations, and 3 cases due to edema. A total of 329 infants were ultimately included in the final analysis. The complete patient selection process is illustrated in Figure 2.

This comparative study included 329 preterm infants undergoing PICC placement. Baseline characteristics were well‐balanced between the one‐time placement group (*n* = 230) and the multiple placement group (*n* = 99), with no statistically significant differences in demographic, perinatal, or procedural parameters. Cesarean section delivery was comparable between groups (76% vs. 79%, χ^2^ = 0.15, *p* = 0.69), and gestational age showed no significant difference (29.01 ± 2.83 vs. 29.61 ± 3.18 weeks, *t* = 0.65, *p* = 0.51). The right lower limb was the preferred PICC insertion site in both groups (93% vs. 95%, χ^2^ = 0.16, *p* = 0.52), indicating consistent clinical practice and standardized procedures for complex neonatal interventions. Insertion personnel were standardized using numeric coding (1–6), with results showing no significant impact of operator variability on PICC placement outcomes (*p* = 0.58) (Table 1).

**TABLE 1:** Baseline characteristics of patients.

Characteristics	PICC one‐time placement group (*n* = 230)	PICC multiple placement group (*n* = 99)	χ^2^ */Z*	*p* value
Delivery mode			0.15	0.69
Cesarean section	175 (76%)	78 (79%)		
Vaginal delivery	55 (24%)	21 (21%)		
Sex			0.02	0.89
Female	97 (42%)	41 (41%)		
Male	133 (58%)	58 (59%)		
Gestational age (w)	29.01 ± 2.83	29.61 ± 3.18	0.65	0.51
1 min Apgar score	7.40 ± 2.16	7.57 ± 1.89	−0.64	0.52
5 min Apgar score	8.93 ± 1.54	9.04 ± 1.24	−0.65	0.51
Admission weight (kg)	1.10 ± 0.28	1.09 ± 0.26	0.01	0.94
Admission length (cm)	35.79 ± 3.53	35.75 ± 3.01	0.01	0.92
Admission head circumference (cm)	26.14 ± 2.59	26.13 ± 2.42	0.01	0.98
Catheter insertion site			0.16	0.51
Left lower limb	16 (7%)	5 (5%)		
Right lower limb	214 (93%)	94 (95%)		
Catheter insertion personnel			0.30	0.48
1	61 (26.5%)	36 (36.4%)		
2	41 (17.8%)	19 (19.2%)		
3	56 (24.3%)	18 (18.2%)		
4	23 (10.0%)	9 (9.1%)		
5	22 (9.6%)	9 (9.1%)		
6	27 (11.7%)	8 (8.1%)		
Time interval between admission and insertion (d)	1.81 ± 2.70	2.14 ± 2.56	−1.02	0.31

Data are presented as mean ± standard deviation (mean ± SD) or number (percentage) [N (%)]. Categorical variables were analyzed using χ^2^ test; continuous variables were analyzed using an independent *t*‐test. Continuous variables are reported as mean (standard deviation) or median (interquartile range). Insertion personnel were standardized by numeric coding (1–6) to minimize operational variability between personnel.

### 3.2. Anthropometric Characteristics and Vascular Selection in PICC

In this study of VLBWI undergoing PICC placement, anthropometric characteristics at insertion revealed a body weight of (1.09 ± 0.26) kg, body length of (35.81 ± 3.34) cm, and head circumference of (26.15 ± 2.51) cm. PICC‐related measurements showed a surface‐measured length of (19.67 ± 2.28) cm and an actual insertion length of (19.39 ± 2.22) cm. The PS‐PTC distance was (5.73 ± 1.62) cm, and the PTC‐CT distance was (13.67 ± 1.53) cm, providing precise anatomical references for PICC insertion. Regarding vessel selection, the great saphenous vein emerged as the primary puncture vessel, accounting for 92% (304/329) of cases, followed by the small saphenous vein at 7% (23/329), with the dorsal foot vein used in only 1% (2) of insertions, highlighting the clinical preference for the great saphenous vein in VLBWI PICC placement (Table 2).

**TABLE 2:** Anthropometric and procedural characteristics of PICC placement.

Characteristics	Mean + SD/N (%)
Anthropometric measurements	
Body weight (kg)	1.09 ± 0.26
Body length (cm)	35.81 ± 3.34
Head circumference (cm)	26.15 ± 2.51
PICC insertion measurements	
Surface‐measured length (cm)	19.67 ± 2.28
Actual insertion length (cm)	19.39 ± 2.22
PS‐PTC distance (cm)	5.73 ± 1.62
PTC‐CT distance (cm)	13.67 ± 1.53
Vessel selection	
Great saphenous vein	304 (92%)
Small saphenous vein	23 (7%)
Dorsal foot vein	2 (1%)

Abbreviations: PS‐PTC distance, puncture site to popliteal transverse crease distance; PTC‐CT distance, popliteal transverse crease to catheter tip distance; surface‐measured length, distance from puncture site to xiphoid process measured with infant supine.

### 3.3. Correlation Analysis of PICC Insertion Depth and Anthropometric Parameters in VLBWI

**FIGURE 3:**
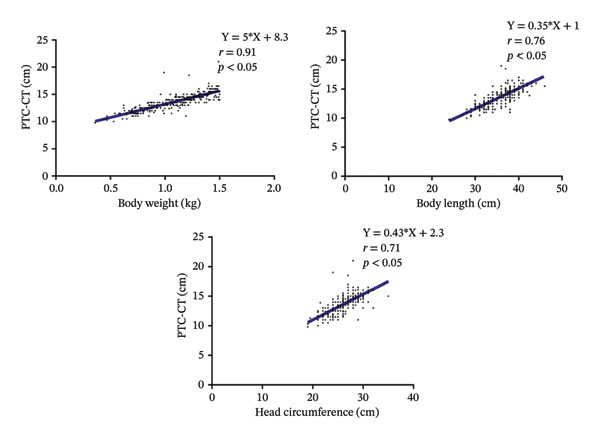
Correlation analysis between anthropometric parameters and PICC insertion depth in VLBWI. Correlation between body weight and catheter tip distance (*r* = 0.91, *p* < 0.05). Correlation between body length and catheter tip distance (*r* = 0.76, *p* < 0.05). Correlation between head circumference and catheter tip distance (*r* = 0.71, *p* < 0.05).

Pearson correlation analysis was performed to systematically evaluate the relationship between the distance from the popliteal transverse crease to the catheter tip and key anthropometric indicators in VLBWI. The results revealed a very strong positive correlation between body weight and catheter tip distance (*r* = 0.91, *p* < 0.05), a strong positive correlation with body length (*r* = 0.76, *p* < 0.05), and a strong positive correlation with head circumference (*r* = 0.71, *p* < 0.05) (Figure 3). These findings indicate that body weight, length, and head circumference are critical determinants of PICC insertion depth, with body weight being the strongest predictor. This discovery provides a foundation for more precise individualized positioning strategies in neonatal PICC placement, potentially reducing associated complication risks.

### 3.4. Regression Equations for PICC Insertion Depth in VLBWI

#### 3.4.1. Linear Regression Prediction Models for PICC Insertion Depth in VLBWI

Linear regression models were developed to analyze the relationship between PICC insertion depth and anthropometric parameters in VLBWI, with popliteal transverse crease to catheter tip distance (PTC‐CT) as the dependent variable. The weight‐based model showed the best predictive performance (*R* ^2^ = 0.83, *F* = 746.25, *p* < 0.001) with the regression equation: PTC‐CT distance (cm) = 5.0 × body weight (kg) + 8.31. In this equation, 8.31 is the regression intercept (constant term), representing the estimated baseline PTC‐CT distance derived statistically from the study population when body weight is held at zero; it serves as a calibration constant rather than a clinically interpretable value at the individual level. The coefficient of five is the unstandardized regression slope, indicating that for each 1 kg increase in body weight, the predicted PTC‐CT distance increases by 5 cm, reflecting the strong and proportional association between neonatal weight and vascular anatomy.

The total PICC insertion depth is calculated according to the following rule:

Total insertion depth (cm) = PTC‐CT distance (cm) + PS‐PTC distance (cm), when the puncture site is distal to (below) the first popliteal crease; total insertion depth (cm) = PTC‐CT distance (cm) − PS‐PTC distance (cm), when the puncture site is proximal to (above) the first popliteal crease.

For example, in a 1 kg infant (predicted PTC‐CT = 5 × 1 + 8.31 = 13.31 cm): if the puncture site is 7 cm below the popliteal crease (PS‐PTC = +7 cm), total insertion length = 13.31 + 7 = 20.31 cm; if the puncture site is 1 cm above the popliteal crease (PS‐PTC = −1 cm), total insertion length = 13.31 − 1 = 12.31 cm (Table 3).

**TABLE 3:** Linear regression equations predicting PICC insertion depth.

Variable	*F*	*b*	*SE*	*β*	*R*	*R* ^2^	95% CI		*t*	*p*
							Upper	Lower		
BW	746.25	8.31	0.17	5.00	0.91	0.83	4.47	5.12	27.31	< 0.001
BL	452.97	1.13	0.02	0.35	0.76	0.58	0.32	0.38	21.28	< 0.001
HC	331.77	2.33	0.02	0.43	0.71	0.50	0.38	0.48	18.21	< 0.001

*Note:* In each regression equation, *b* represents the intercept (constant term) and β represents the unstandardized regression coefficient (slope). For the weight‐based model, the intercept *b* = 8.31 is a statistical calibration constant, and the slope *β* = 5.00 indicates that PTC‐CT distance increases by 5 cm per 1 kg increase in body weight.

Abbreviations: BL, body length; BW, body weight; HC, head circumference; R, correlation coefficient; *R* ^2^, coefficient of determination; SE, standard error; 95% CI, 95% confidence interval for slope coefficient.

#### 3.4.2. Multiple Regression Prediction Models for PICC Insertion Depth in VLBWI

Multiple regression models incorporating several anthropometric parameters were developed to improve prediction accuracy. Model IV (including body weight, length, and head circumference) demonstrated the best predictive performance (*R* ^2^ = 0.84, *F* = 257.36, *p* < 0.001). Model II (weight + head circumference) also performed well (*R* ^2^ = 0.83, *F* = 375.37, *p* < 0.001). Model III (length + head circumference) showed relatively weaker predictive capability (*R* ^2^ = 0.79, *F* = 272.63, *p* < 0.001). All multiple regression models were statistically significant, but the improvement in predictive ability was modest, suggesting that body weight remains the primary predictor of PICC insertion depth (Table 4). In each multivariate model, the intercept *b* represents the estimated PTC‐CT distance when all predictor variables equal zero, serving as a statistical baseline. The regression coefficients for body weight, body length, and head circumference reflect the independent contribution of each variable to the predicted PTC‐CT distance after controlling for the others. Model IV (intercept *b* = 6.01) showed the highest *R* ^2^ (0.84), with body weight contributing the dominant effect (coefficient = 3.74), followed by body length (0.07) and head circumference (0.04). Clinicians using any multivariate model should apply the same ± rule as described above: adding the PS‐PTC distance when the puncture site is distal to the popliteal crease, and subtracting it when proximal.

**TABLE 4:** Multiple regression equations predicting PICC insertion depth.

Model	Variables	*F*	*b*	*β*			*R* ^2^	*p*
				BW	BL	HC		
Model I	BW + BL	271.17	6.54	3.97	0.08	—	0.70	< 0.001
Model II	BW + HC	375.37	7.57	4.46	—	0.05	0.83	< 0.001
Model III	BL + HC	272.63	−0.05	—	0.24	0.19	0.79	< 0.001
Model IV	BW + BL + HC	257.36	6.01	3.74	0.07	0.04	0.84	< 0.001

*Note:* Regression equations: Model I: *Y* = 6.54 + 3.97 × BW + 0.08 × BL; Model II: *Y* = 7.57 + 4.46 × BW + 0.05 × HC; Model III: *Y* = −0.05 + 0.24 × BL + 0.19 × HC; Model IV: *Y* = 6.01 + 3.74 × BW + 0.07 × BL + 0.04 × HC.

Abbreviations: BL, body length (cm); BW, body weight (kg); HC, head circumference (cm); *R* ^2^, coefficient of determination.

### 3.5. Accuracy Assessment of PICC Catheter Length Prediction Formulas

**FIGURE 4:**
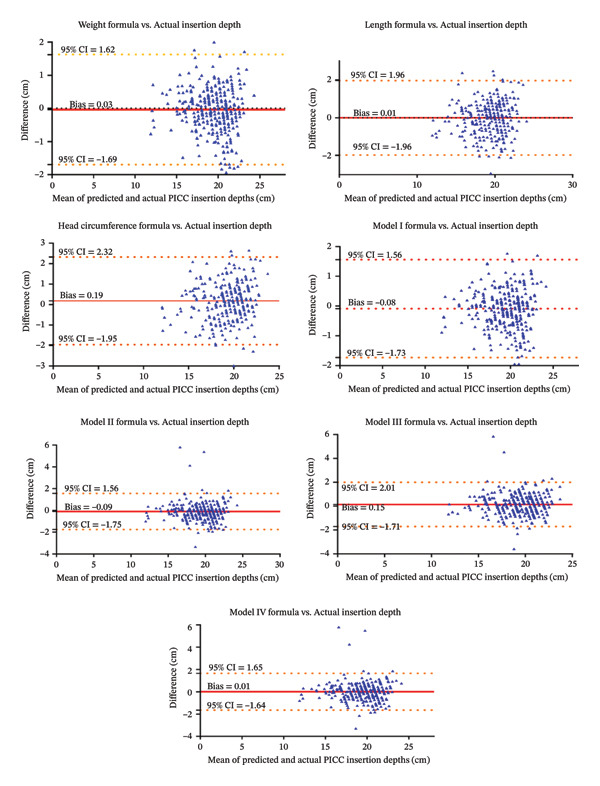
Bland–Altman analysis of PICC catheter insertion length predictions.

Bland–Altman analysis was performed to evaluate the accuracy of prediction formulas. The weight‐based model showed slight systematic bias (0.03 cm) with 95% limits of agreement from −1.69 to 1.62 cm. The length‐based model demonstrated minimal bias (0.01 cm) with 95% limits of agreement from −1.96 to 1.96 cm. The head circumference model exhibited more pronounced systematic bias (0.19 cm) with 95% limits of agreement from −1.95–2.32 cm. Among multiple regression models, Model I showed slight negative bias (−0.08 cm, 95% CI: −1.73 to 1.56 cm), Model II had a bias of −0.09 cm (95% CI: −1.75 to 1.56 cm), Model III showed bias of 0.15 cm (95% CI: −1.71–2.01 cm), and Model IV demonstrated near‐zero bias (0.01 cm, 95% CI: −1.64–1.65 cm). Most measurements were distributed within the 15–25 cm range with symmetric distribution around the zero‐difference line. Paired *t*‐tests confirmed that the weight‐based, length‐based models and Model IV showed no systematic bias (*p* > 0.05), while the head circumference model and some multiple regression models demonstrated significant bias (*p* < 0.05) (Table 5, Figure 4).

**TABLE 5:** Accuracy assessment of PICC catheter length prediction models.

PICC prediction model	Catheter depth (cm)	Residual value (cm)	*t*	*p* value
Actual correct PICC insertion length (cm)	19.39 ± 2.22	—	—	—
Weight‐based prediction (cm)	19.43 ± 2.31	0.03 ± 0.85	0.561	0.575
Length‐based prediction (cm)	19.40 ± 2.17	0.01 ± 1.01	0.024	0.981
Head circumference‐based prediction (cm)	19.21 ± 2.10	−0.19 ± 1.09	−3.127	0.002
Model I (BW + BL) prediction (cm)	19.48 ± 2.26	0.09 ± 0.83	1.932	0.054
Model II (BW + HC) prediction (cm)	19.49 ± 2.25	0.10 ± 0.83	2.127	0.034
Model III (BL + HC) prediction (cm)	19.25 ± 2.20	−0.15 ± 0.94	−2.853	0.005
Model IV (BW + BL + HC) prediction (cm)	19.39 ± 2.25	−0.01 ± 0.82	−0.165	0.869

*Note:* Data are presented as mean ± standard deviation. *p* values < 0.05 were considered statistically significant. Model I: predictive model based on BW and BL; Model II: predictive model based on BW and HC; Model III: predictive model based on BL and HC; Model IV: predictive model based on BW, BL, and HC.

Abbreviation: PICC, peripherally inserted central catheter.

## 4. Discussion

### 4.1. Association Between Admission Weight and PICC Insertion Depth in VLBWI

This study focused on the relationship between anthropometric parameters and lower extremity PICC insertion length among VLBWI. Employing a prospective cohort design, we consecutively enrolled a substantial sample of 329 VLBWI undergoing lower limb PICC placement over one year. The large sample size and consecutive case inclusion ensure robust representativeness and reliability of the findings. A notable methodological feature was the anatomical landmark selection—the first popliteal crease—which minimized measurement bias. We systematically quantified the relationship between anthropometric parameters and catheter length and constructed both univariate and multivariate linear regression prediction models.

Results demonstrated a strong positive correlation between body weight and PICC insertion depth (*R* ^2^ = 0.83, *p* < 0.001), yielding the regression equation: Ideal PTC‐CT distance (cm) = 5 × body weight (kg) + 8.31, where 8.31 is the regression intercept and five is the slope coefficient representing a 5 cm increase in PTC‐CT distance per 1 kg increase in body weight. The length‐based model showed correlation (*R* ^2^ = 0.58, *p* < 0.001) with the equation: Ideal catheter tip depth (cm) = 0.35 × body length (cm) ± 1.13, where 1.13 is the regression intercept and 0.35 is the slope coefficient representing a 0.35 cm increase in PTC‐CT distance per 1 cm increase in body weight. The head circumference‐based model demonstrated weaker correlation (*R* ^2^ = 0.50, *p* < 0.001). Multivariate regression analysis revealed that Model IV (weight + length + head circumference) achieved the highest coefficient of determination (*R* ^2^ = 0.84, *p* < 0.001).

Bland–Altman analysis showed that the weight‐based model had a mean prediction bias of 0.03 cm with 95% limits of agreement ranging from −1.69 to 1.62 cm. The length‐based model demonstrated minimal bias of 0.01 cm with 95% limits of agreement from −1.96 to 1.96 cm. Model IV showed bias of 0.01 cm with 95% limits of agreement from −1.64 to 1.65 cm, all indicating excellent clinical concordance. Paired *t*‐tests confirmed no systematic bias for the weight‐based, length‐based models, and Model IV (*p* > 0.05). Collectively, these findings suggest that anthropometric parameter‐based prediction formulas offer intuitive, scientific, and individualized references for PICC length selection in VLBWI, markedly improving first‐attempt success rates and standardizing practice while minimizing complication risks.

### 4.2. Comparison With Previous Studies and Interpretation of Differences

Our study demonstrated a significant positive correlation between body weight and lower extremity PICC insertion length in VLBWI. This finding aligns with the results of Kim [15], who performed a retrospective analysis of 138 preterm and low birth weight infants and reported that body weight could reliably predict lower limb PICC length (*R* ^2^ = 0.846). The strong correlation established in our regression model (*R* ^2^ = 0.83) is consistent with Kim’s findings, confirming the feasibility and utility of using weight as a prediction factor for catheter length. Kim’s study, however, was retrospective with a slightly smaller sample size and did not standardize the puncture site at the first popliteal crease, whereas our prospective cohort adopted this anatomical marker, thereby minimizing localization errors. In contrast, a study by Agrawal [18] in India, which enrolled 85 VLBWI, reported that traditional surface measurement methods had limited accuracy for predicting PICC length, resulting in higher tip misplacement rates. Unlike our study, Agrawal did not include weight as a predictive variable; nor did they standardize the anatomical landmark, which contributed to greater measurement variability. Furthermore, Luister [16] developed multivariate formulas, but most existing studies remain retrospective and lack unified puncture sites or anatomical markers, which limits their generalizability. Mechanistically, body weight reflects the overall developmental status of neonates and directly correlates with vascular length and distribution, making it ideal for generating quantitative prediction models that improve catheter placement success and reduce complication rates [2, 19].

Our study, by standardizing the anatomical reference and employing a large‐sample prospective design with Bland–Altman analysis, ensures greater methodological rigor and clinical applicability. He [8] reported that real‐time ultrasound guidance could theoretically improve placement accuracy and reduce errors for VLBWI, but its uptake was limited by operator experience and resource constraints, highlighting the pragmatic value and accessibility of our weight‐based, landmark‐guided approach. In contrast, studies like Li [7] used retrospective multicenter cohorts, with nonstandardized puncture sites and potential operator‐dependent bias, while Bahoush [4] focused primarily on multiple vascular access modes in pediatric patients rather than strictly VLBWI and lower extremity PICC prediction. Our prospective cohort further enhances the credibility and relevance of weight as a predictive parameter. Collectively, our study not only corroborates the feasibility and advantages of individualized, weight‐ and landmark‐based prediction for lower extremity PICC placement but also provides a methodological and theoretical template for future multicenter studies on neonatal vascular access.

### 4.3. Clinical Implications and Future Perspectives

This study demonstrates significant clinical value in the context of lower extremity PICC placement for VLBWIs. By establishing a predictive formula for catheter length that integrates neonatal body weight, length and head circumference with a standardized anatomical landmark (the first popliteal crease), the study provides clinicians with a simple, objective, and accurate reference for PICC insertion length, which helps reduce repeated punctures and associated complications, thereby improving the first‐attempt success rate. This approach not only optimizes procedures in neonatal intensive care, but also has the potential to lower infection rates and decrease healthcare resource consumption. Compared to previous methods that relied on surface measurements or empirical estimates, our use of a large, prospective cohort and uniform puncture site selection enhances the scientific rigor and generalizability of the predictive model, advancing the standardization of PICC placement [20]. The formula is especially valuable in settings where resources are limited and real‐time ultrasound guidance is not routinely available, making it highly applicable and promotable across different regions. Clinically, it is recommended that practitioners prioritize an individualized, weight‐based prediction formula for VLBWI to enhance safety and efficiency.

This study offers several notable strengths. Firstly, the prospective cohort design ensured the integrity of data collection and systematic follow‐up, accurately reflecting real‐world clinical practice in lower extremity PICC placement for VLBWIs. The inclusion of a large, consecutive sample greatly enhanced both the representativeness of the study population and the statistical power of our findings. Secondly, our strict standardization of the first popliteal crease as the puncture landmark minimized measurement errors caused by operator subjectivity, thus improving the reliability of the results. Standardized procedures for data collection ensured precise recording of key variables such as body weight and effectively reduced information bias. In terms of data analysis, we developed a linear regression model and systematically assessed the accuracy and agreement of the prediction formula using the Bland–Altman method, which added further rigor and practical relevance to our conclusions. The comprehensive application of multiple statistical approaches increased the study’s reliability and clinical value. Together, these methodological strengths establish a solid evidence base for future research and clinical practice in this field [21].

### 4.4. Study Limitations

This study has several limitations. First, due to the inclusion and exclusion criteria, only eligible VLBWIs were enrolled, excluding those with severe congenital malformations, significant cardiopulmonary disease, or venous anomalies. This may limit the applicability of our findings to these special subpopulations, and caution is warranted when extrapolating the results. Second, as a single‐center study conducted exclusively among Chinese patients, the generalizability and external validity of our conclusions are limited, particularly when applying them across different regions or ethnicities. Third, the marked predominance of right lower limb insertions (308/329, 93.6%) precluded a statistically valid laterality‐stratified analysis. Although Bland–Altman analysis confirmed near‐zero systematic bias across the combined cohort, suggesting that any laterality‐related differences in vascular path length did not meaningfully affect prediction accuracy at the population level, formal comparative analysis between left and right lower limb insertions was not feasible given the limited number of left‐sided cases. Future multicenter studies with larger and more balanced samples should examine whether separate laterality‐specific formulas are warranted.

## 5. Conclusion

This study established anthropometric parameter‐based prediction formulas for lower extremity PICC insertion length in VLBWI, with body weight demonstrating the strongest predictive value (*R* ^2^ = 0.83) and the multivariate model (weight + length + head circumference) achieving the best overall performance (*R* ^2^ = 0.84). These formulas provide clinicians with a simple, objective, and individualized reference for PICC length selection, with direct potential to reduce catheter tip malposition, minimize repeated punctures, lower infection risk, and improve first‐attempt success rates. The approach is particularly applicable in resource‐limited NICUs and primary healthcare institutions where real‐time ultrasound guidance is unavailable or cost‐prohibitive, supporting equitable access to safe vascular access practice across different regions and healthcare systems. Furthermore, the standardization of the first popliteal crease as an anatomical landmark has implications for promoting consistent, reproducible PICC insertion practices in neonatal nursing. Future multicenter studies enrolling more diverse patient populations are warranted to further validate and extend the generalizability of these predictive formulas.

## Funding

This work was supported by the Guangdong Provincial High‐level Clinical Key Specialty Project (project number: SZGSP009); the Shenzhen Natural Science Foundation Plan Project (project numbers: JCYJ20250604191408012); Shenzhen Science and Technology Program (project numbers: JCYJ20230807120213026 and JCYJ20240813115035045); Research Fund of Shenzhen Maternity & Child Healthcare Hospital (project number: FYB2022004); Shenzhen “Sanming” Project of Medicine (project number: SZSM202211001); the Key Laboratory for Pregnancy Disorders and Maternal and Child Health of Shenzhen (project number: ZDSYS20230626091559006); Maternal and Child Health Research Project (Maternal and Infant Nutrition and Health Special Program) of the National Center for Women and Children’s Health, National Health Commission (2025FYH21).

## Conflicts of Interest

The authors declare no conflicts of interest.

## Data Availability

The data that support the findings of this study are available on request from the corresponding author. The data are not publicly available due to privacy or ethical restrictions.
